# 5-Lipoxygenase Activating Protein (FLAP) Dependent Leukotriene Biosynthesis Inhibition (MK591) Attenuates Lipid A Endotoxin-Induced Inflammation

**DOI:** 10.1371/journal.pone.0102622

**Published:** 2014-07-15

**Authors:** Wen-Feng Fang, Ivor S. Douglas, Chin-Chou Wang, Hsu-Ching Kao, Ya-Ting Chang, Chia-Cheng Tseng, Kuo-Tung Huang, Huang-Chih Chang, Meng-Chih Lin

**Affiliations:** 1 Division of Pulmonary and Critical Care Medicine, Department of Internal Medicine, Kaohsiung Chang Gung Memorial Hospital, Chang Gung University College of Medicine, Kaohsiung, Taiwan; 2 Department of Respiratory Care, Chang Gung University of Science and Technology, Chiayi Campus, Chiayi, Taiwan; 3 Division of Pulmonary Sciences and Critical Care Medicine, University of Colorado, Denver and Denver Health Medical Center, Denver, Colorado, United States of America; Fundação Oswaldo Cruz, Brazil

## Abstract

The Lipid A moiety of endotoxin potently activates TLR-4 dependent host innate immune responses. We demonstrate that Lipid-A mediated leukotriene biosynthesis regulates pathogen-associated molecular patterns (PAMP)-dependent macrophage activation. Stimulation of murine macrophages (RAW264.7) with *E. coli* 0111:B4 endotoxin (LPS) or Kdo2-lipid A (Lipid A) induced inflammation and Lipid A was sufficient to induce TLR-4 mediated macrophage inflammation and rapid ERK activation. The contribution of leukotriene biosynthesis was evaluated with a 5-lipoxygenase activating protein (FLAP) inhibitor, MK591. MK591 pre-treatment not only enhanced but also sustained ERK activation for up to 4 hours after LPS and Lipid A stimulation while inhibiting cell proliferation and enhancing cellular apoptosis. Leukotriene biosynthesis inhibition attenuated inflammation induced by either whole LPS or the Lipid A fraction. These responses were regulated by inhibition of the key biosynthesis enzymes for the proinflammatory eicosanoids, 5-lipoxygenase (5-LO), and cyclooxygenase-2 (COX-2) quantified by immunoblotting. Inhibition of leukotriene biosynthesis differentially regulated TLR-2 and TLR-4 cell surface expression assessed by flow cytometry, suggesting a close mechanistic association between TLR expression and 5-LO associated eicosanoid activity in activated macrophages. Furthermore, MK591 pre-treatment enhanced ERK activation and inhibited cell proliferation after LPS or Lipid A stimulation. These effects were regulated in part by increased apoptosis and modulation of cell surface TLR expression. Together, these data clarify the mechanistic association between 5-lipoxygenase activating protein-mediated leukotriene biosynthesis and 5-LO dependent eicosanoid metabolites in mediating the TLR-dependent inflammatory response after endotoxin exposure typical of bacterial sepsis.

## Introduction

Sepsis commonly causes severe organ failures including acute lung injury (ALI) and results in high mortality and substantial morbidity. Despite advances in critical care, there are no specific therapies available to treat sepsis and hospital mortality remains as high as 44.5% in patients admitted to Asian intensive care units [1]. Similarly, the mortality rate amongst septic shock patients randomized to the placebo arm of the recent Prospective Recombinant Human Activated Protein C Worldwide Evaluation in Severe Sepsis and Septic Shock (PROWESS-SHOCK) study was 32.7% at 90 days [2].

Lipopolysaccharide (LPS), a constituent of the outer membrane of Gram-negative bacteria (GNB) [3] is recognized as a key to the pathogenesis of GNB-associated sepsis. Recognition of bacterial pathogen-associated molecular patterns (PAMPs) such as LPS, by macrophage toll-like receptors is a key component of host defences against infection by Gram-negative bacteria [4]. LPS consists of O-antigen, core, and lipid A which has been considered responsible for the pyrogenic activity of endotoxins [5], [6]. TLR-4 is the primary receptor ligand for LPS. However, a limitation of using native LPS for mechanistic studies of endotoxin activity is its large molecular size and micro-heterogeneity, especially in the length and composition of its terminal glycan chains. Macromolecular LPS binds monocyte cell-surface expressed PAMPS including TLR-4 and -2, resulting in MyD88-dependent signaling and downstream inflammatory cascade activation via phospoho-activation of IκB kinase (IKK)-β and MAP kinases (MAPKs). This in turn mediates NF-κB and AP-1 dependent nuclear transcription of pro-inflammatory cytokines including IL-1 and TNFα [7].

Studies of receptor-ligand specificity and downstream pro-inflammatory signaling cascades have potentially been confounded by non-TLR-4 binding by macromolecular LPS. We previously described that compared with macromolecular LPS, the Lipid A fraction of LPS induces a discrete MAPK activation (ERK activation) in murine acute lung injury [8].

Eicosanoid metabolites are important inflammatory mediators that have been implicated in the pathogenesis of sepsis-associated ALI. Two major pathways of eicosanoid metabolism include cyclooxygenase (COX) (producing prostaglandins, thromboxanes, and prostacyclin) and 5-lipoxygenase (5-LO) (producing leukotrienes). The synthesis of leukotrienes from substrate arachidonic acid is initiated by 5-LO in concert with 5-lipoxygenase-activating protein (FLAP). FLAP enhances the ability of 5-LO to interact with its substrate. When 5-LO is activated, it relocates from the nucleoplasm to the outer or inner nuclear membrane [9].

Eicosanoid pathway regulators have been studied in pre-clinical models [10], [11] and prospective human studies of sepsis and lung injury [12]. Inhibitors of COX metabolites, while promising in animal models, were ineffective in large clinical trials of sepsis-associated lung failure [12]. Several leukotriene mediators have been demonstrated to be pathogenic in inflammatory diseases including sepsis [9] and sepsis associated ALI [13]. In a murine cecal ligation and puncture model comparing wild-type (WT) and 5-LO knockout mice, Monteiro and coworkers implicated 5-lipoxygenase products in the pathogenesis of sepsis induced lung injury [10]. Further, lung inflammation was significantly and comparably reduced in both 5-LO knockout mice and WT mice treated with a pharmacologic 5-LO inhibitor. However, the mechanisms by which leukotriene biosynthesis inhibition dampens endotoxin-induced inflammation is unclear. Whether this effect is restricted to Lipid-A/TLR-4 mediated inflammation or less specific macromolecular LPS/PAMP signaling has also not been reported.

The present study was conducted to determine the specificity of Lipid A-mediated leukotriene-dependent biosynthesis in the regulation of PAMP-dependent macrophage activation as a model of sepsis.

## Materials and Methods

### Materials

*E. coli* 0111:B4 endotoxin was purchased from Sigma Chemical (St. Louis, MO). Kdo2-lipid A was purchased from Avanti Polar Lipids (Alabaster, AL). A specific 5-lipoxygenase activating protein (FLAP) inhibitor, MK591, was provided by Merck Sharp & Dohme Corp. (Whitehouse Station, NJ).

### Murine Macrophage culture

RAW264.7 macrophages (Food Industry Research and Development Institute, Taiwan) were cultured in Dulbecco’s modified Eagle’s medium with 4 mM L-glutamine adjusted to contain 3.7 g sodium bicarbonate and supplemented with 4.5 g/L glucose, 10% fetal bovine serum (FBS), penicillin (100 units/ml), and streptomycin (100 µg/ml) (Life Technologies, Gaithersburg, MD), in a humidified atmosphere of 95% air and 5% CO2 at 37°C. Adherent cells (70% confluence) were studied after overnight 1% FBS DMEM incubation.

### Cytokine ELISA

Immunoreactive TNF-α and MIP-2 of cell culture medium were quantified using commercially available ELISA kits (R&D Systems, Minneapolis, MN), according to manufacturer’s instructions.

### Western blot analysis

Western blots to detect levels of phosphorylated and total ERK, 5-lipoxygenase (5-LO), Cyclooxygenase-2 (COX-2) were performed. Details are presented in Methods S1.

### Cell viability assay

To determine cell viability, Cell Proliferation Reagent WST-1 (Roche Diagnostics, Mannheim, Germany) was used according to the manufacturer’s specifications. The assay is based on the enzymatic cleavage of the tetrazolium salt WST-1 to formazan by cellular mitochondria dehydrogenases present in viable cells. 5×10^5^ RAW264.7 cells/well were seeded into 96-well plates in 1% FBS DMEM and incubated in 37°C overnight, and the intervention groups were pre-treated with 5-lipoxygenase-activating protein (FLAP) inhibitor, MK591, at a final concentration of 0, 10, 25, 50 and 100 µM, respectively 30 minutes prior to stimulation. LPS or Lipid A were added to the wells at a final concentration of 100 ng/ml. After 4 hr incubation, the fraction of viable cells was determined using µ-Quant ELISA Reader (Bio-Tek Instruments, USA) at 450 nm wavelength.

### In vitro apoptosis analysis

The effect of MK591 on murine macrophage apopotosis was analyzed by in situ terminal deoxynucleotidyl transferase dUTP nick end labeling (TUNEL) staining. Methods are detailed in the online supplemental (Methods S1).

### Flow-cytometry analysis for TLR-2 and TLR-4

RAW264.7 culture cells were washed with PBS first. For surface marker staining, cells were measured by simultaneously staining for 30 minutes with directly conjugated monoclonal antibodies, anti-TLR-2 (Anti-Human/Mouse CD282 (TLR-2) PE, eBioscience, San Diego, CA, USA) and TLR-4 mAb (Anti-Mouse TLR4/MD-2 Complex PE-Cy7, eBioscience, San Diego, CA, USA) or with isotype control antibody (eBioscience, San Diego, CA, USA). Immunofluorescence analysis performed on a Beckman Coulter Cytomics FC500 Flow Cytometry. All marker expressions were gated by the peak of control group and presented by percentage of total cells.

### Statistical analysis

For each experimental condition, the entire group was prepared and studied at the same time. Each intervention group was repeated two/or three to seven time. Data are presented as means ± SEM for each experimental group. GraphPad Prism 5 was used for graphic plotting. SigmaStat v3.11 was used for one-way analysis of variance, the Tukey-Kramer continuity correction was applied to correct for multiple comparisons.

## Results

### Effects of LPS and Lipid-A on RAW264.7 cell viability

RAW264.7 cell viability was 101.38%±7.24%(LPS group) or 96.11±6.09 (Lipid A group) after incubation for 4 hours with LPS or Lipid-A 100 ng/ml and was comparable to vehicle control (100%±0.0%; p = 0.87 compared to LPS group, p = 0.59 compared to Lipid A group).

### MK591 blocks LPS and Lipid-A induced macrophage inflammatory mediator release

**Figure 1:**
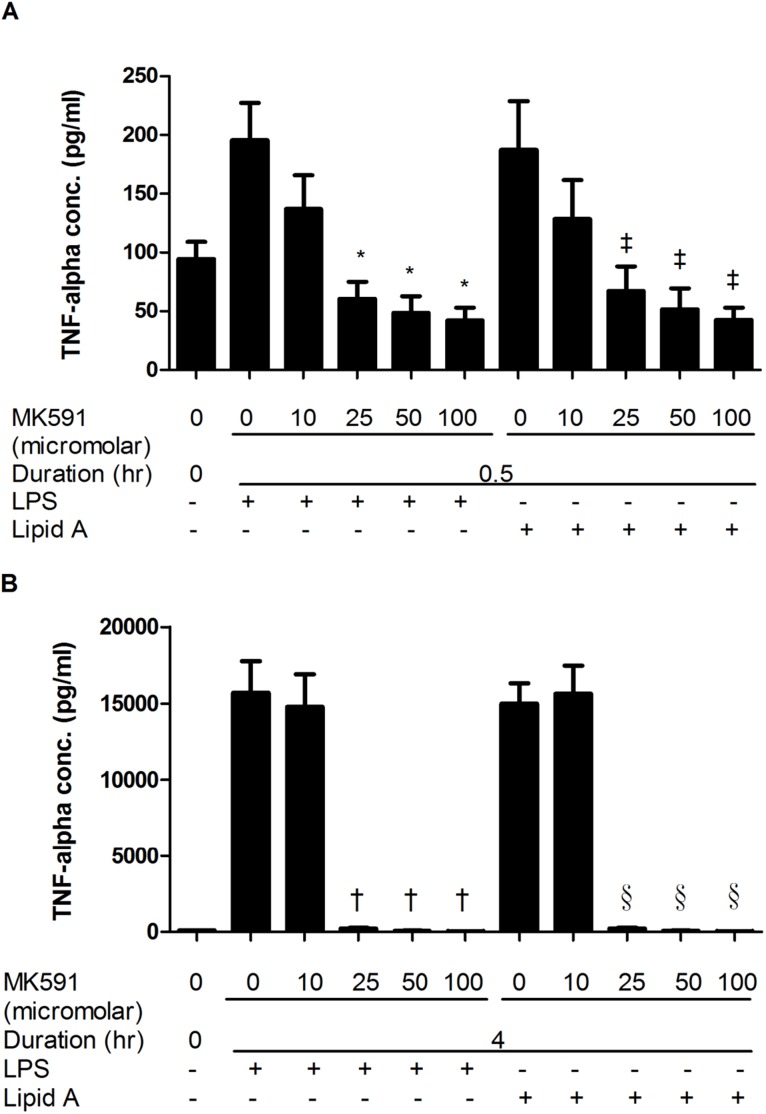
MK591 pre-treatment of RAW264.7 cells reduced LPS and Lipid A induced TNF-α secretion. RAW264.7 cells were pre-treated for 30 minutes with MK591 or vehicle following by LPS or Lipid A stimulation for 0.5 hr (A) and 4 hr (B). Conditioned media were collected and TNF-α levels analyzed by ELISA. Quantitative data are presented as means ± SEM from 6–7 independent experiments. *p<0.01 and ^†^p<0.001 compared to LPS-treated cells and ^‡^p<0.01 and ^§^p<0.001 compared to Lipid A-treated cells.

**Figure 2:**
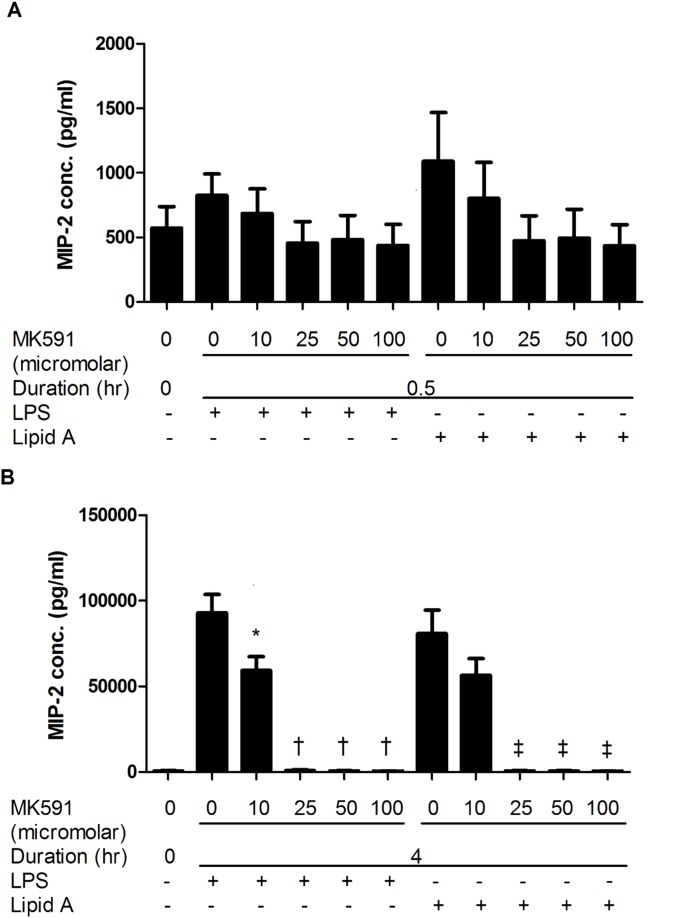
MK591 pre-treatment of RAW264.7 cells reduced LPS and Lipid A induced MIP-2 secretion. RAW264.7 cells were pre-treated for 30 minutes with MK591 or vehicle followed by LPS or Lipid A stimulation for 0.5 hr (A) and 4 hr (B). Quantitative data are presented as means ± SEM from 6–7 independent experiments. *p<0.05 and ^†^p<0.001 compared to LPS-treated cells and ^‡^p<0.01 compared to Lipid A-treated cells.

Dose and time response studies were performed with the pharmacological inhibitor MK591 to determine the role of FLAP, the 5-LO activating protein, on LPS and Lipid A-induced TNF-α (Fig. 1) and MIP-2 (Fig. 2) secretion. There was a significant reduction in TNF-α release after MK591 treatment at both time points evaluated (Fig. 1A and B) (at 0.5 h, mean ± SEM, LPS 195.57±31.70 vs. LPS-MK591 100 µM 42.17±10.61, p<0.01; Lipid A 187.10±41.61 vs. Lipid A-MK591 100 µM 42.34±10.74, p<0.01; n = 7) (at 4 h, mean ± SEM, LPS 15691.27±2082.27 vs. LPS-MK591 100 µM 40.84±6.24, p<0.001; Lipid A 14987.45±1337.81 vs. Lipid A-MK591 100 µM 36.75±6.35, p<0.001; n = 6). There was no difference in MIP-2 release at 30 minutes after MK591 treatment (Fig. 2A), but a significant reduction was observed after 4 hours (Fig. 2B) (mean ± SEM, LPS 92984.77±10693.19 vs. LPS-MK591 100 µM 438.67±108.67, p<0.001; Lipid A 80829.38±13722.52 vs. Lipid A-MK591 100 µM 436.30±104.19, p<0.01; n = 6). Based on these observations, the threshold dose of 25 µM MK591 was selected for pre-treatment in subsequent studies.

### Leukotriene biosynthesis inhibitor enhances ERK activation after Lipid A and LPS stimulation

To determine the mechanism of leukotriene biosynthesis inhibition on LPS and Lipid A mediated cytokine expression protein expression ERK (p44/42), 5-lipoxygenase (5-LO) and cyclooxygenase 2(COX-2) were quantified in cell lysates by immunoblotting.

**Figure 3:**
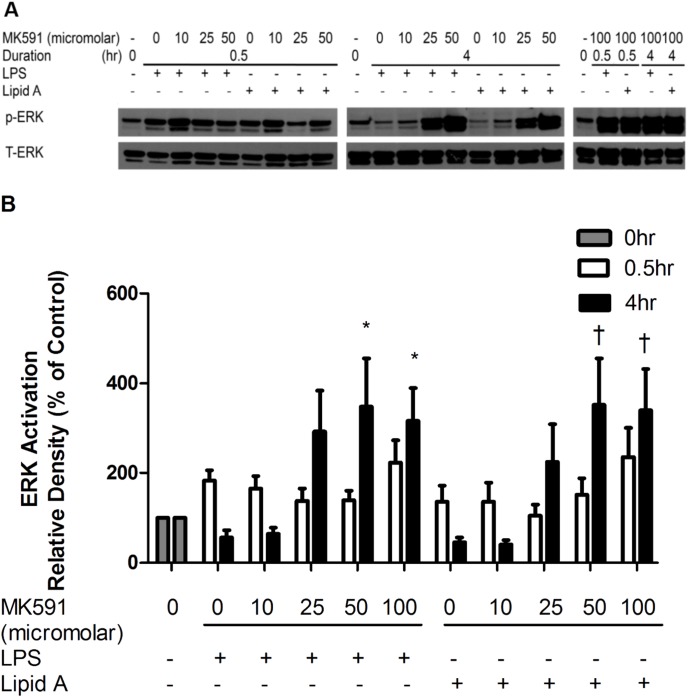
MK591 pre-treatment enhanced ERK activation in RAW264.7 cells stimulated by LPS and Lipid A. (A) RAW264.7 cells were pre-treated for 30 minutes with MK591 or vehicle followed by LPS or Lipid A stimulation for 0.5 hr or 4 hr. Activation of ERK was evaluated by immunoblotting of RAW264.7 cell lysates. Representative immunoblots of 5 independent experiments are presented. Total ERK was quantified as an internal control. (B) Quantitative data are presented as means ± SEM from 5 independent experiments. *p<0.05 compared to LPS-treated cells and ^†^p<0.05 compared to Lipid A-treated cells.

Modest, transient pERK activation was induced by LPS treatment at 30 minutes but returned to pre-treatment expression levels after 4 hr when compared with vehicle control (Figure 3A). MK591 pretreatment not only enhanced the level of pERK activation but also significantly prolonged ERK activation at 4 h after LPS or Lipid A stimulation. This pattern of potentiated phospho-activation was particularly pronounced 4 h after LPS and Lipid A stimulation (mean ± SEM, LPS 56.89±15.86 vs. LPS-MK591 100 µM 316.14±73.44, p<0.05; Lipid A 45.82±10.45 vs. Lipid A-MK591 100 µM 339.63±92.72, p<0.05; n = 5–7) (figure 3). By contrast, MK591 treatment alone inhibited ERK expression in a dose dependent fashion in RAW264.7 cells (figure S1).

### Leukotriene biosynthesis inhibitor attenuates 5-lipoxygenase expression after Lipid A or LPS stimulation

**Figure 4:**
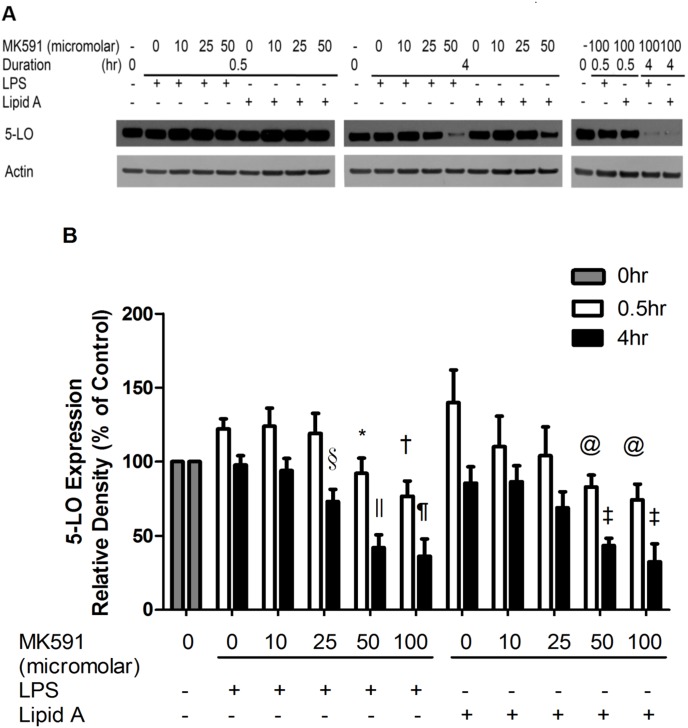
MK591 pre-treatment inhibits 5-LO expression in RAW264.7 cells stimulated by LPS and Lipid A. (A) RAW264.7 cells were pre-treated for 30 minutes with MK591 or vehicle followed by LPS or Lipid A stimulation for 0.5 hr and 4 hr. Expression of 5-LO were evaluated by immunoblotting of RAW264.7 cell lysates. Actin density was quantified as internal control. Representative immunoblots of 5 independent experiments are presented. (B) Quantitative expression is the mean ± S.E.M. of 5 independent experiments. At 0.5 hr, *p<0.05 and ^†^p<0.01 compared to LPS-treated cells, ^‡^p<0.05 compared to Lipid A-treated cells; as at 4 hr, ^§^p<0.05, ^¶^p<0.01 and ^||^p<0.001 compared to LPS-treated cells, ^@^p<0.01 compared to Lipid A-treated cells.

Expression of the key biosynthesis enzyme of proinflammatory leukotrienes, 5-lipoxygenase (5-LO), was down-regulated in a dose dependent fashion by MK591 pre-treatment at 0.5 h (mean ± SEM, LPS 122.1±6.86 vs. LPS-MK591 100 µM 76.59±10.34, p<0.01; Lipid A 140.02±22.08 vs. Lipid A-MK591 100 µM 74.35±10.64, p<0.05; n = 6–7) and 4 h (mean ± SEM, LPS 97.644±6.63 vs. LPS-MK591 100 µM 36.02±11.96, p<0.01; Lipid A 85.51±11.01 vs. Lipid A-MK591 100 µM 32.33±12.35, p<0.01; n = 6–7) (figure 4). MK591 treatment alone inhibited 5-LO expression in a dose dependent fashion in RAW264.7 cells (figure S2).

**Figure 5:**
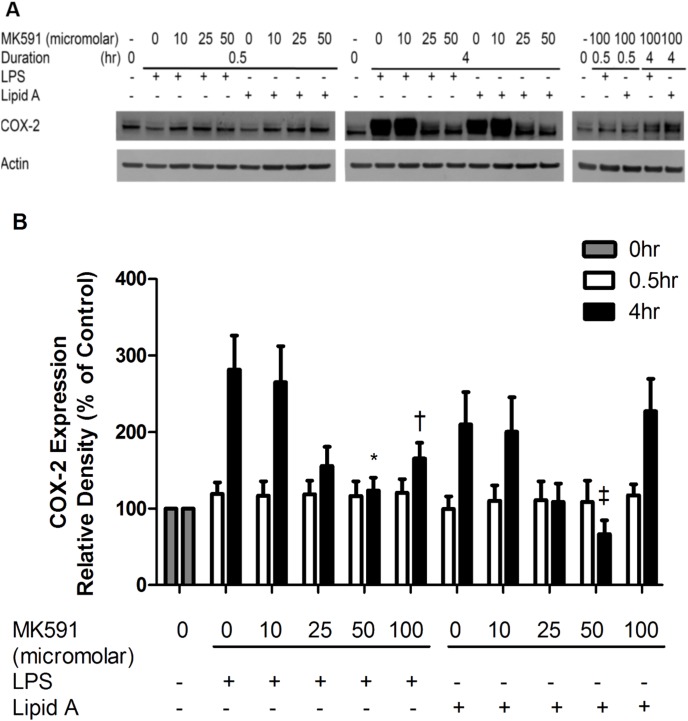
MK591 pre-treatment inhibits COX-2 expression in RAW264.7 cells stimulated by LPS and Lipid A. (A) RAW264.7 cells were pre-treated for 30 minutes with MK591 or vehicle followed by LPS or Lipid A stimulation for 0.5 hr and 4 hr. Expression of COX-2 were examined by immunoblotting using RAW264.7 cell lysates. Actin density was quantified as internal control. Quantitative expression is the mean ± S.E.M. of 5 independent experiments. (B) Quantitative expression is the mean ± S.E.M. of 5 independent experiments. *p<0.05 compared to LPS-treated cells and ^†^p<0.05 compared to Lipid A-treated cells.

In contrast the inducible regulator of proinflammatory prostaglandins cyclooxygenase 2 (COX-2), was up-regulated 4 hours (but not 30 minutes) after LPS and Lipid A stimulation. After LPS stimulation for 4 hours, a significant reduction in COX-2 expression was observed with the highest concentration of MK591 (25–100 uM). With Lipid A stimulation, 50 uM of MK591 was sufficient to induce a significant reduction in COX-2 expression (figure 5). MK591 treatment alone inhibited COX-2 expression in a dose dependent fashion in RAW264.7 cells (figure S3).

### Leukotriene biosynthesis inhibitor impedes macrophage proliferation independently of LPS or Lipid A stimulation

**Figure 6:**
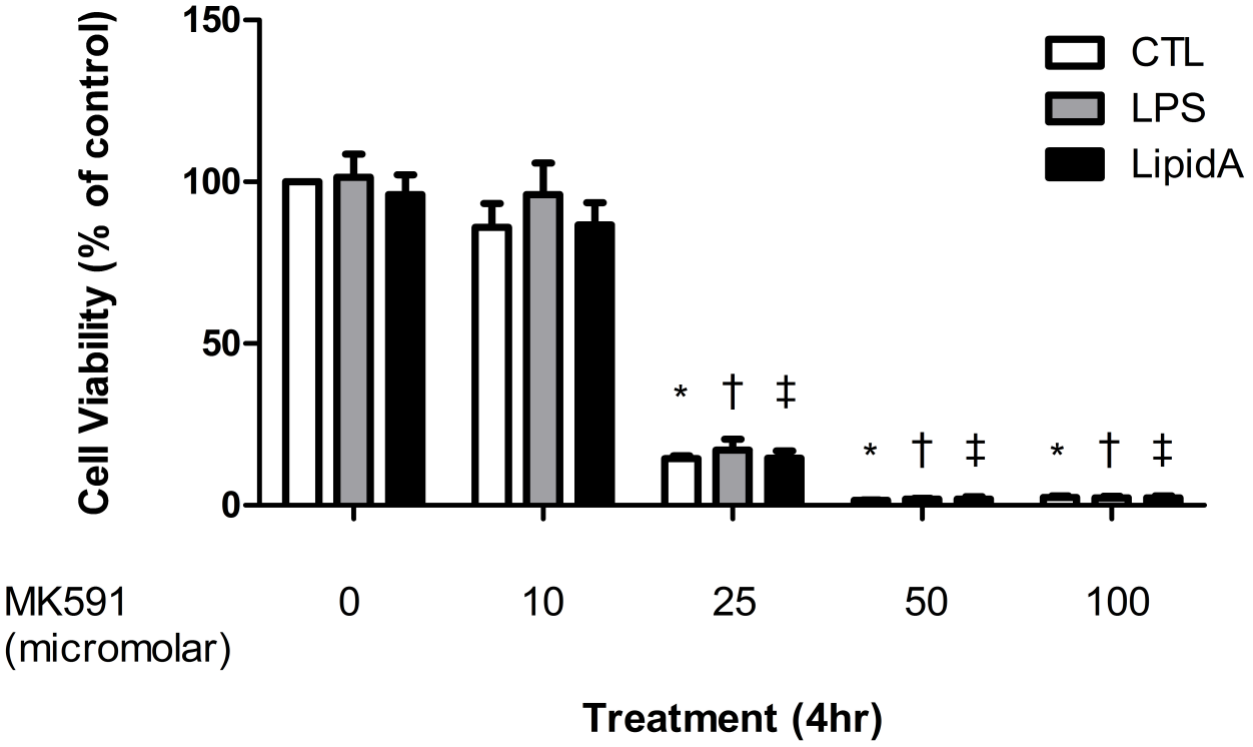
MK591 pre-treatment inhibits RAW264.7 cell proliferation using WST-1 proliferation assay. RAW264.7 cells were pre-treated for 30 minutes with MK591 or vehicle followed by LPS or Lipid A stimulation for 0.5 hr and 4 hr. Cell viability was analyzed by WST-1 and quantitative assessment of proliferation is presented as the mean ± S.E.M. *p<0.001 compared to non-treated control cells, ^†^p<0.001 compared to LPS-treated cells and ^‡^p<0.001 compared to Lipid A-treated cells.

To investigate the effect of leukotriene biosynthesis inhibition on murine macrophage proliferation, cells were seeded in 96 well-plates and grown to 70% confluence. Cell proliferation was determined by WST-1 assay. Proliferation was consistently inhibited in a dose-dependent fashion after MK0591 pre-treatment and independent of LPS or Lipid A stimulation (mean ± SEM, Control 100±0.00 vs. Control-MK591 100 µM 2.45±0.52, p<0.001, LPS 101.38±7.24 vs. LPS-MK591 100 µM 2.33±0.54, p<0.01; Lipid A 96.11±6.09 vs. Lipid A-MK591 100 µM 2.30±0.63, p<0.01, n = 3, figure 6).

### Leukotriene biosynthesis inhibitor induces apoptosis after Lipid A or LPS stimulation

**Figure 7:**
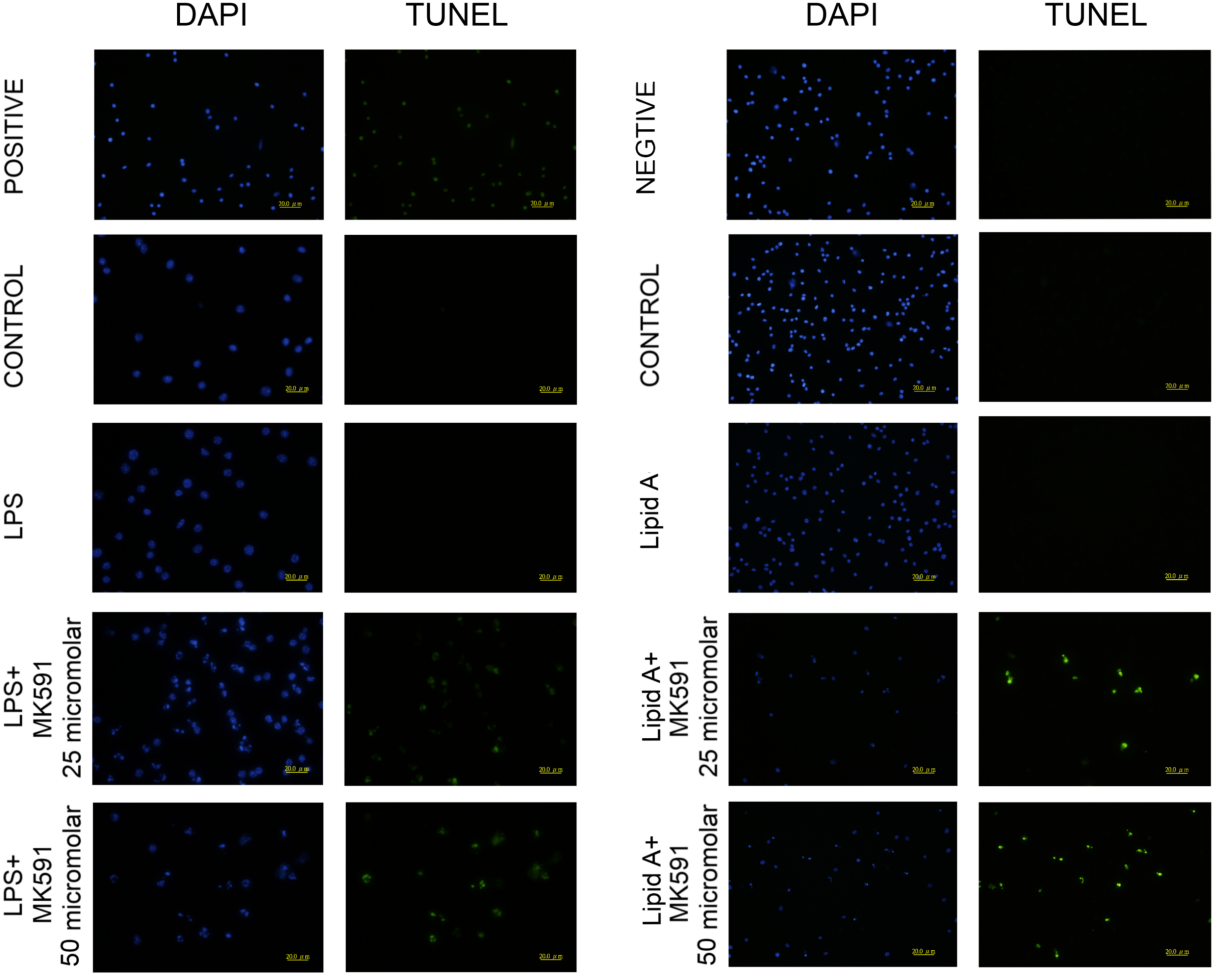
MK591 pretreatment mediates dose-dependent increases in RAW264.7 apoptosis assessed by TUNEL staining. RAW264.7 cells were pre-treated for 30 minutes with MK591 or vehicle followed by LPS or Lipid A stimulation for 0.5 hr and 4 hr. Apoptotic nuclei were detected with FITC (green fluorescence), and all nuclei were counterstained with DAPI (blue fluorescence). Apoptotic cell number increased in a MK591 dose-dependent fashion. Original magnification, ×400.

To further determine the effect of MK591 on murine macrophage proliferation and determine if cell death was an important contributor, the presence of apoptosis was measured by TUNEL assay. MK591 pre-treatment induced murine macrophages apoptosis in a dose dependent fashion both in macrophages exposed to Lipid A or LPS (figure 7). MK591 treatment alone also induced apoptosis in RAW264.7 cells (figure S4).

### Leukotriene biosynthesis inhibitor modulated cell surface receptor TLR expression

We next investigated the influence of MK591 on Toll-like Receptor (TLR-2 and -4) expression by use of flow cytometry. RAW264.7 cells were pre-treated with 25 µM MK591 followed by LPS (100 ng/ml) or Lipid A (100 ng/ml) stimulation.

Cell surface TLR-2 expression was rapidly and persistently up-regulated from 0.5 h through 4 h after either LPS or Lipid A (mean ± SEM, Control 49.72±0.23 vs. LPS 60.20±4.03, p<0.05, Control 49.72±0.23 vs Lipid A 60.12±3.19, p<0.05, n = 6–7). However, MK591 pretreatment attenuated significantly TLR-2 expression 4 hours post stimulation compared with vehicle pre-treated cells (mean ± SEM, LPS 60.20±4.03 vs. LPS-MK591 25 µM 29.12±4.43, p<0.001, and Lipid A 60.12±3.19 vs. Lipid A-MK591 25 µM 30.52±2.99, p<0.001, n = 6–7, figure S5).

Cell surface TLR4 expression was also significantly enhanced and sustained 4 hours post stimulation LPS or Lipid-A stimulation compared with vehicle pre-treated control cells (mean ± SEM, Control 50.22±0.13 vs. LPS 41.86±0.43 p<0.001, Control 50.22±0.13 vs. Lipid A 32.18±4.76, p<0.05).

By contrast, pre-treatment with MK-591 further enhanced cell surface TLR-4 expression. (LPS 41.86±0.43 vs. LPS-MK591 25 µM 63.95±6.03, p<0.05; Lipid A 32.18±4.76 vs. Lipid A-MK591 25 µM 66.01±6.06, p<0.01, n = 4, figure S6).

## Discussion

We have demonstrated the regulatory effects of leukotriene biosynthesis on LPS mediated inflammation in macrophages. We established that the FLAP inhibitor, MK591 attenuated inflammation induced by either whole LPS or the Lipid A fraction of LPS. Furthermore, we demonstrated that leukotriene biosynthesis inhibition enhanced ERK activation and impededs cell proliferation after LPS or Lipid A stimulation. The likely mechanisms regulating this effect of leukotriene biosynthesis inhibition are increased apoptosis and differential modulation of cell surface receptor TLR expression (figure S7).

We previously reported elevated eicosanoid expression (by HPLC and ELISA) in total lung homogenate of mice with Lipid A or LPS induced lung injury. 5-LO protein production was amplified in mice treated with LPS [14]. Leukotrienes contribute to endotoxin-induced impairment of hypoxic pulmonary vasoconstriction [11]. In addition, inhibition of 5-LO-activating protein protects against experimental ventilator induced lung injury [15]. 5-LO knockout mice and wild type mice treated with a pharmacologic 5-LO inhibitor were significantly protected from lung inflammation and injury [10]. These data support the potential feasibility of pharmacological leukotriene biosynthesis inhibition as a potential therapy for endotoxin mediated sepsis.

Leukotrienes are lipid mediators with pro-inflammatory [16], bronchoconstrictor and vasoactive effects that are attractive therapeutic targets in the treatment of pulmonary inflammation including asthma and sepsis-associated lung injury [17]. The synthesis of leukotrienes from substrate arachidonic acid is initiated by 5-lipoxygenase in concert with nuclear membrane 5-lipoxygenase–activating protein (FLAP) [9]. For the present studies our focus was limited to the membrane bound MAPEG (membrane-associated proteins in eicosanoid and glutathione metabolism) superfamily proteins, specifically FLAP.

The Toll-like receptor (TLR) family of cell surface receptors transduce pathogen-associated molecular pattern (PAMP) signalling by infectious agents including bacteria and initiate host-innate immune responses [18]. Toll-like receptor 4 (TLR-4), is central to the host cellular inflammatory response to LPS [19]–[23]. However, although Gram-negative bacterial infection is primarily regulated by TLR-4, TLR-2 activation is thought to play an import modulating role. TLR-4-induced IFN-gamma production increases TLR-2 sensitivity and drives Gram-negative sepsis in mice [24]. Consistent with this, we demonstrated that both LPS and Lipid A stimulation promote surface TLR-2 expression suggesting that the Lipid A moiety is required for this effect.

Lipid A, a fraction of LPS, which has been considered responsible for the pyogenic activity of endotoxins, was sufficient in our study to induce *in* *vitro* macrophage activation comparable in magnitude to whole LPS. These findings are consistent with our previous observations that structural differences between whole LPS and the lipid A moiety may account for the striking differences in ERK activation patterns in whole lung but not isolated murine macrophages [8]. Kinases other than ERK such as JNK, p38 did not exhibit differential activation between LPS and Lipid A. Therefore, we focus on ERK in this study. In the context of whole organ lung injury pathogenesis, cells other than macrophages, such as lung structural cells including pulmonary microvascular endothelial cells and alveolar pneumocytes that express TLR-4 and respond to LPS, may account for the differential ERK activation in acute lung injury.

Mendis and colleagues reported that MK591 had a modest inhibitory effect on TNF-α expression induced by staphylococcal enterotoxin B (from Gram positive bacteria) in human peripheral blood mononuclear cells. They also demonstrated that inhibitors of the lipoxygenase pathway were able to block Staphylococcal enterotoxin B (SEB) induced ERK activation [25]. However, our studies show that MK591 pre-treatment enhanced and potentiated pERK activation. Although this may be due to TLR pathway activation by other PAMPs, an alternative explanation may be that inhibition of leukotriene biosynthesis with MK591 repressed mitogen-associated kinase phosphatase (MKP) enzyme activity [26] thus potentiating ERK activation and consequently LPS-mediated inflammation. The striking autocrine enhancement in cell-surface TLR-4 expression after MK591 incubation potentially amplifying signal transduction may offer a further explanation.

Depending on stimulus and cell type, ERK activity can mediate different antiproliferative events, such as apoptosis [27]. We showed that pre-incubation with the leukotriene biosynthesis inhibitor decreased cell number independently of Lipid A or LPS stimulation. Cell viability was unaffected by LPS or Lipid A stimulation while MK591 treatment impeded macrophage proliferation in a dose-dependent fashion. MK591 also enhanced to a comparable level, Lipid A and LPS mediated apoptosis. These data explain in part why MK591 attenuated Lipid A or LPS-induced inflammation in macrophages despite increased ERK activity.

MK591 had the effect of attenuating both Lipid A and LPS associated TLR-4 induction and TLR-2 suppression. These data suggest that 5-LO associated eicosanoid activity is necessary for regulating cell surface expression of these PAMP receptors. It is entirely possible that molecular heterogeneity in LPS structure significantly effects differential TLR-2 or TLR-4 receptor engagement and transactivation. This could potentially mediate synergistic or divergent effects on downstream TLR signalling pathways. In future studies, macrophages from TLR-4, -2 or dual knockout mice could yield important mechanistic insights. Our data do not clarify if MK-591 alters receptor expression at the transcriptional or post-translational level. However the rapid induction in expression after 30 minutes of either Lipid A or LPS exposure suggests this is a non-transcriptional event.

The current investigation focused on the role of membrane bound MAPEG (membrane-associated proteins in eicosanoid and glutathione metabolism) superfamily signaling molecules, in particular FLAP. Studies with pharmacological inhibitors such as zileuton specific for 5-LO, the enzymatic target of FLAP may provide additional mechanistic insights. However, while FLAP and 5-LO are functionally associated they have different molecular mechanisms of action and 5-LO antagonists may regulate endotoxin-mediated leukotriene biosynthesis, in a fashion significantly different in magnitude and effect than regulators of FLAP.

## Conclusion

Our study indicates that Lipid A was sufficient to induce TLR4 mediated macrophage inflammation and that rapid ERK MAPK activation was regulated by 5-LO associated leukotriene products. However, MK591 pre-treatment not only enhanced but also sustained ERK activation for up to 4 hours after LPS and Lipid A induced inflammation while inhibiting cell proliferation and enhancing cellular apoptosis. This was associated with inhibition of the key biosynthesis enzymes regulating of proinflammatory leukotrienes and prostaglandins, 5-lipoxygenase (5-LO) and cyclooxygenase 2 (COX-2). Inhibition of leukotriene biosynthesis also differentially regulated TLR-2 and TLR-4 cell surface expression suggesting a close mechanistic association between TLR expression and 5-LO associated eicosanoid activity in activated macrophages.

## Supporting Information

Figure S1 **MK591 treatment enhances ERK activation in RAW264.7 cells with or without stimulated by LPS and Lipid A.** (A) Activation of ERK was examined by immunoblotting using RAW264.7 cell lysates. The representative immunoblot is from one of 5 independent experiments. Total ERK density was used as internal control. (B) Quantitative data are presented as means ± SEM from 5 independent experiments.
(DOCX)

Figure S2 **MK591 treatment inhibits 5-LO expression in RAW264.7 cells with or without stimulated by LPS and Lipid A.** (A) Expression of 5-LO was examined by immunoblotting using RAW264.7 cell lysates. Actin density blots were used as internal control. The representative immunoblot is from one of 5 independent experiments. (B) Quantitative expression is the mean ± S.E.M. of 5 independent experiments.
(DOCX)

Figure S3 **MK591 treatment inhibits COX-2 expression in RAW264.7 cells with or without stimulated by LPS and Lipid A.** (A) Expression of COX-2 was examined by immunoblotting using RAW264.7 cell lysates. Actin density blots were used as internal control. The representative immunoblot is from one of 5 independent experiments (B) Quantitative results expression is the mean ± S.E.M. of 5 independent experiments.
(DOCX)

Figure S4 **To further determine the effect of MK591 on murine macrophage proliferation and determine if cell death is an important contributor, the presence of apoptosis was measured by TUNEL assay.** MK591 induced murine macrophages apoptosis with or without exposure to Lipid A or LPS.
(DOCX)

Figure S5 **MK591 pre-treatment inhibits LPS or Lipid A-induced TLR2 expression on RAW264.7 cell membranes.** (A) (B) (C) Flow cytometry histograms demonstrate reduced TLR2 expression after MK591 pre-treatment and LPS or Lipid A stimulation for 4 hours; expression is gated by the peak of control group. Shaded histogram represents isotype-matched negative control Ab fluorescence; open histogram represents specific Ab staining. (D) LPS and Lipid A treatment induces surface TLR2 expression, but this was inhibited by MK591 pretreatment. TLR2 expression quantitatively represented as mean ± S.E.M. of 6–7 independent experiments. At 0.5 hr, *p<0.05 compared to non-treated control cells and ^†^p<0.05 compared to LPS-treated cells; as at 4 hr, ^‡^p<0.05 compared to non-treated control cells, ^§^p<0.001 compared to LPS-treated cells and ^¶^p<0.001 compared to Lipid A-treated cells. (E) Representative flow cytometry dotplot demonstrate RAW264.7 cells after MK591 pre-treatment with LPS or Lipid A stimulation for 4 hours.
(DOCX)

Figure S6 **MK591 pre-treatment inhibits LPS or Lipid A-induced TLR4 expression on RAW264.7 cell membranes.** (A) (B) (C) Flow cytometry histograms demonstrate increased TLR4 expression after MK591 pre-treatment and LPS or Lipid A stimulation for 4 hours; expression is gated by the peak of control group. Shaded histogram represents isotype-matched negative control Ab fluorescence; open histogram represents specific Ab staining. (D) LPS and Lipid A treatment reduced surface TLR4 expression, but this was reversed by MK591 pretreatment. Expression is quantitatively represented as the mean ± S.E.M. of 4 independent experiments. At 0.5 hr, *p<0.05 compared to non-treated control cells and ^†^p<0.05 compared to Lipid A-treated cells; as at 4 hr, ^‡^p<0.05 and ^§^p<0.001 compared to non-treated control cells, ^¶^p<0.01 compared to non-treated control cells, ^||^p<0.05 compared to LPS-treated cells and ^#^p<0.01 compared to Lipid A-treated cells. (E) Representative flow cytometry dotplot demonstrate RAW264.7 cells after MK591 pre-treatment with LPS or Lipid A stimulation for 4 hours.
(DOCX)

Figure S7 **Model of LPS and Lipid A fraction regulation of inflammation by leukotriene biosynthesis inhibition.** TLR4/MD2 engagement by LPS or the Lipid A fraction of endotoxin initiates macrophage-mediated inflammation (white arrows) with secretion of pro-inflammatory cytokines TNF-α and MIP 2, secretion of eicosanoid mediators and downregulation of cell-surface TLR4 expression. Leukotriene biosynthesis inhibition with MK591 (black arrows) block FLAP dependent 5-LO leukotriene biosynthesis, attenuates inflammation induced by either whole LPS or the Lipid A fraction of LPS in association with enhanced ERK activation and inhibition of cell proliferation. The likely mechanisms regulating this effect of leukotriene biosynthesis inhibition are increased apoptosis and modulation of cell surface receptor TLR expression.
(DOCX)

Methods S1 **Extended Materials and Methods.**
(DOCX)
