# Supplementary figures and images for: 5-Lipoxygenase Activating Protein (FLAP) Dependent Leukotriene Biosynthesis Inhibition (MK591) Attenuates Lipid A Endotoxin-Induced Inflammation
Source: PLoS One. 2014 Jul 15;9(7):e102622. doi: 10.1371/journal.pone.0102622 (PMC4099325; doi:10.1371/journal.pone.0102622)

**Figure S1.**

**
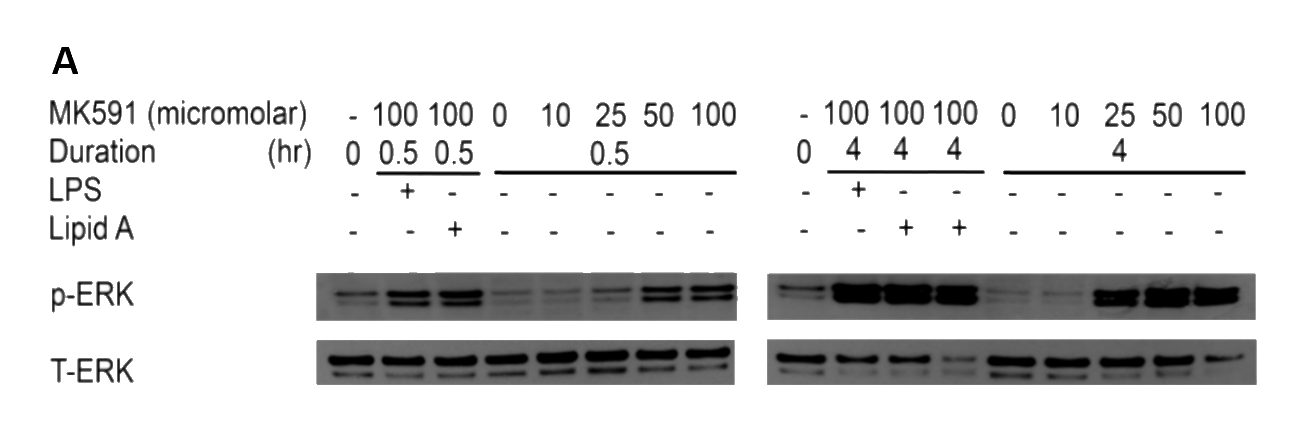
**

**
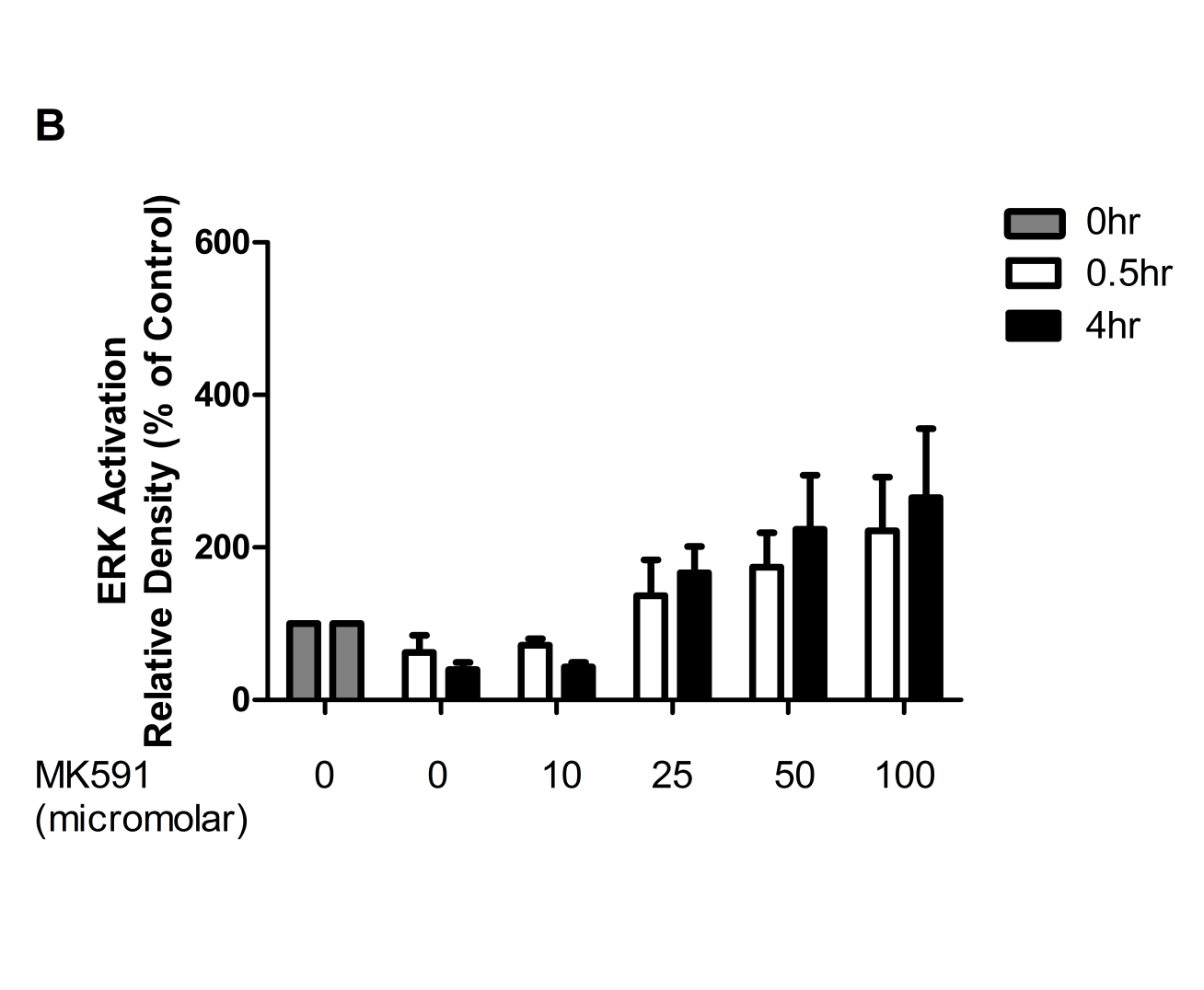
**

Supplement: Figure S1 — MK591 treatment enhances ERK activation in RAW264.7 cells with or without stimulated by LPS and Lipid A. (A) Activation of ERK was examined by immunoblotting using RAW264.7 cell lysates. The representative immunoblot is from one of 5 independent experiments. Total ERK density was used as internal control. (B) Quantitative data are presented as means ± SEM from 5 independent experiments. (DOCX) [file pone.0102622.s001.docx]

**Figure S2.**

**
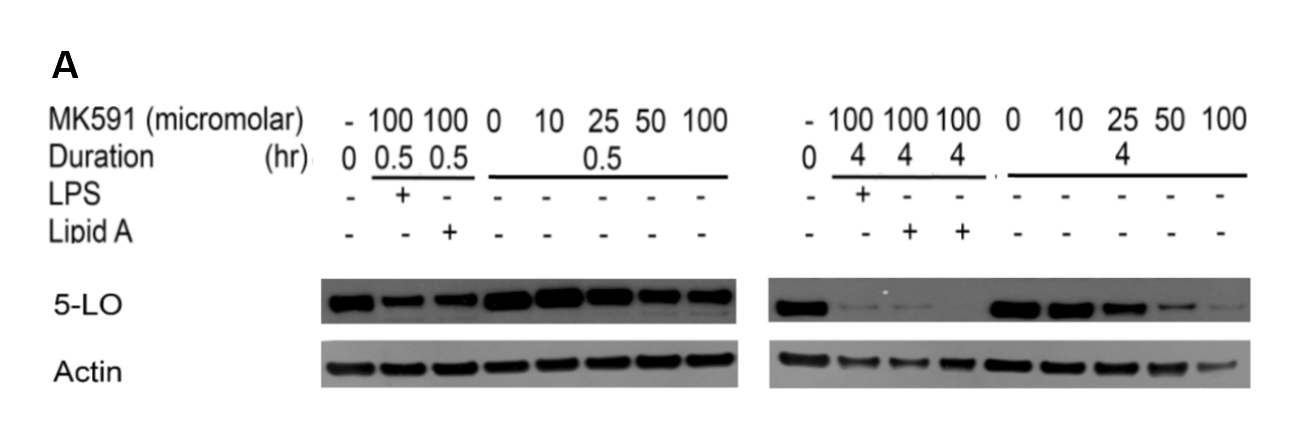
**

**
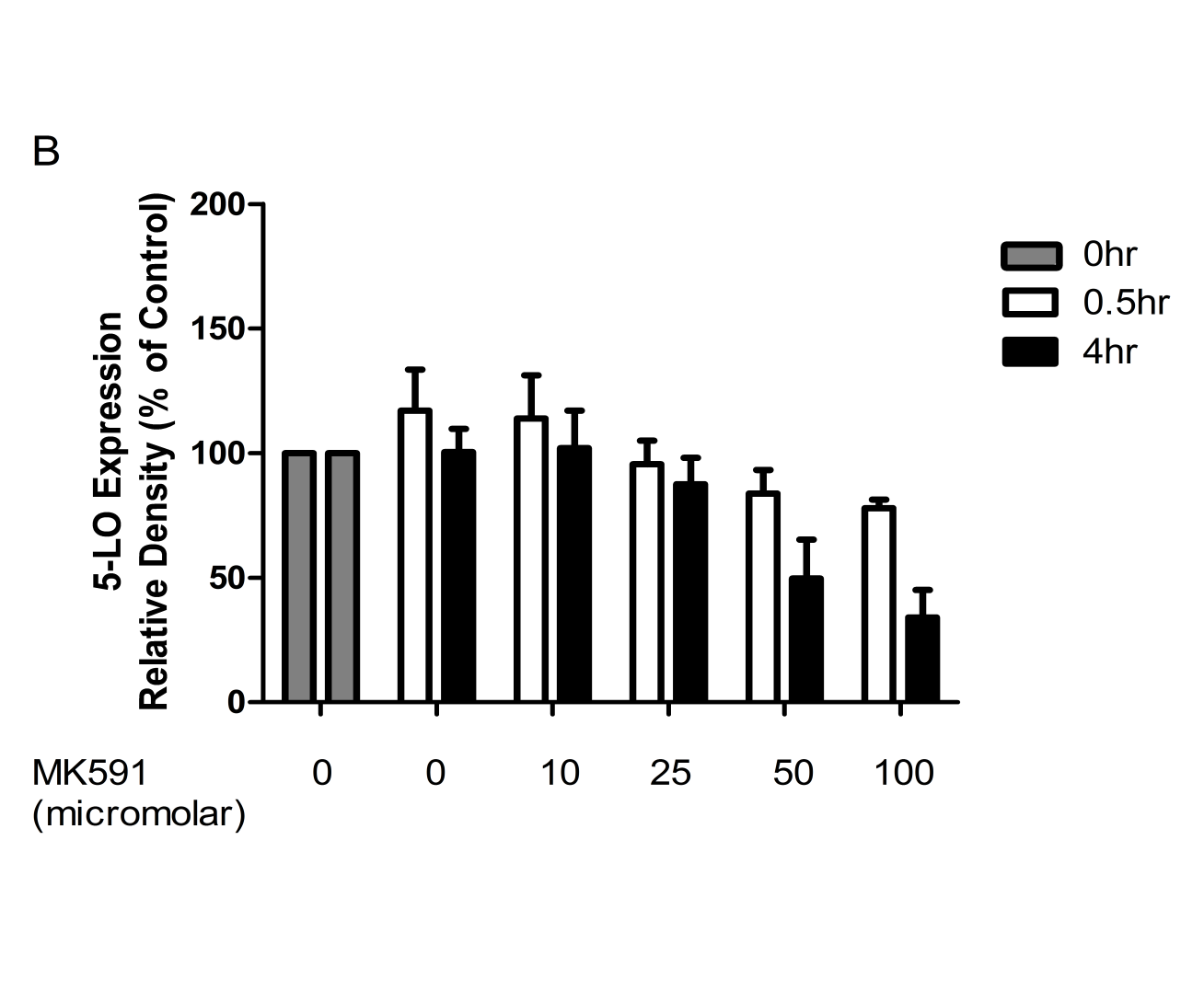
**

Supplement: Figure S2 — MK591 treatment inhibits 5-LO expression in RAW264.7 cells with or without stimulated by LPS and Lipid A. (A) Expression of 5-LO was examined by immunoblotting using RAW264.7 cell lysates. Actin density blots were used as internal control. The representative immunoblot is from one of 5 independent experiments. (B) Quantitative expression is the mean ± S.E.M. of 5 independent experiments. (DOCX) [file pone.0102622.s002.docx]

**Figure S3.**

**
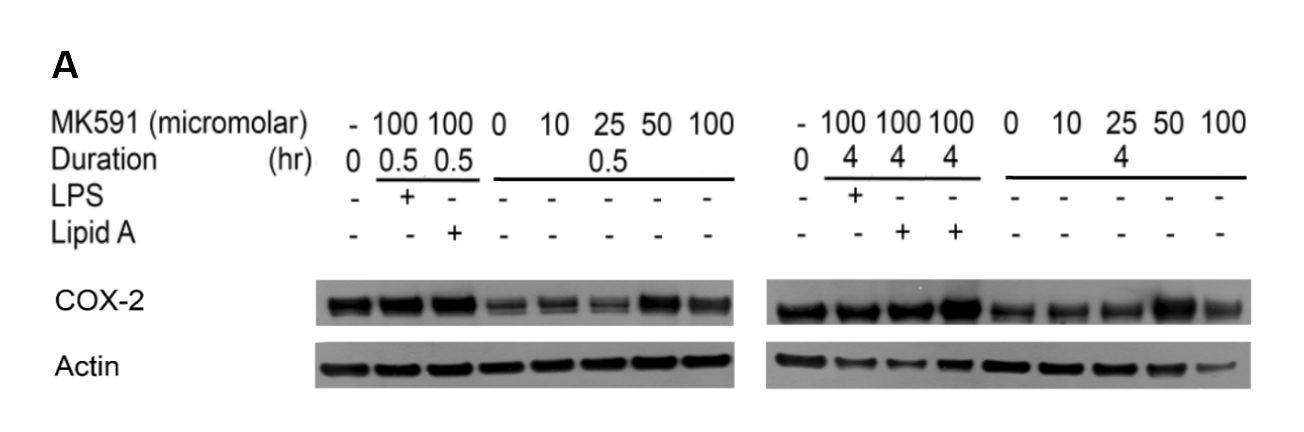
**

**
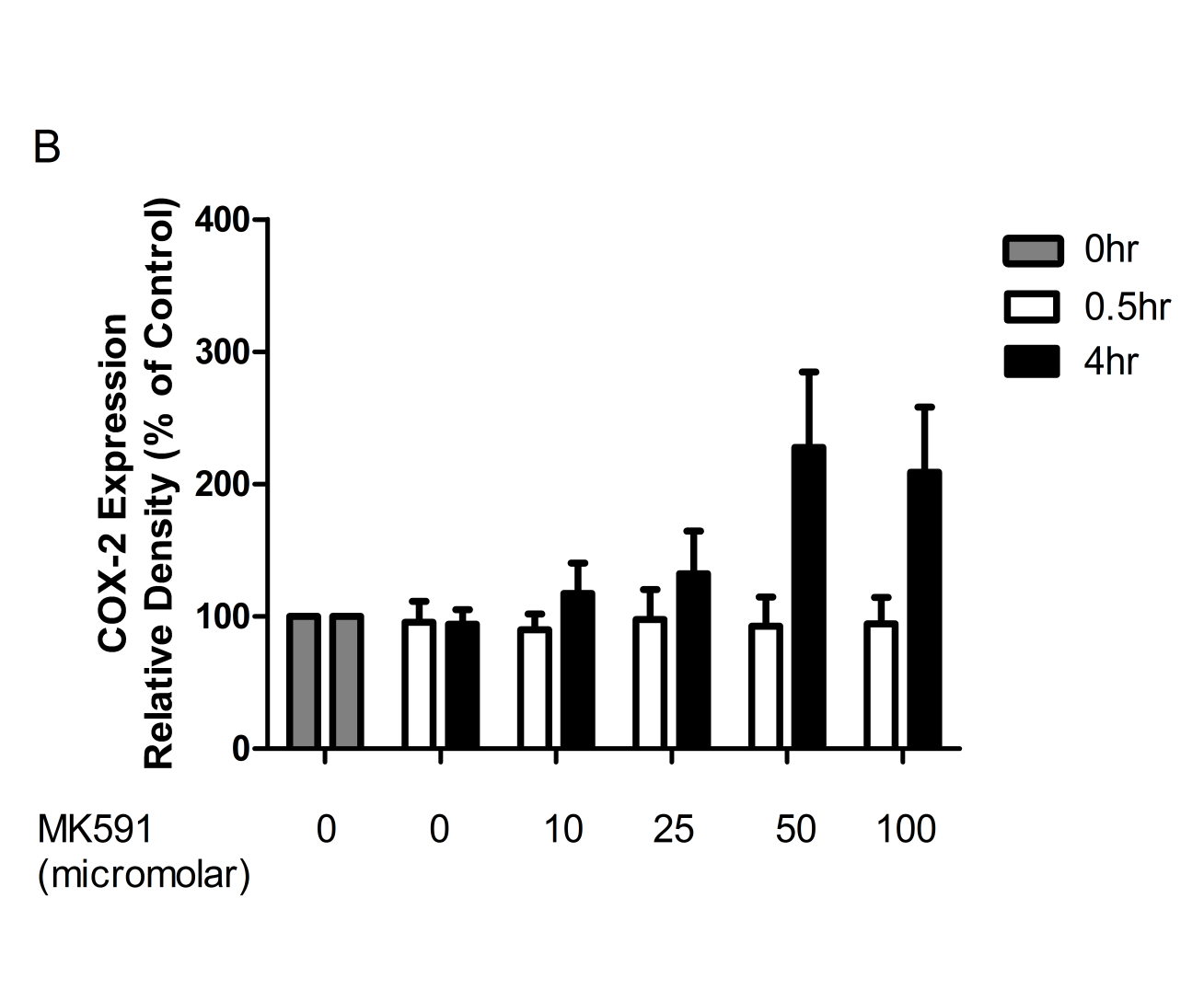
**

Supplement: Figure S3 — MK591 treatment inhibits COX-2 expression in RAW264.7 cells with or without stimulated by LPS and Lipid A. (A) Expression of COX-2 was examined by immunoblotting using RAW264.7 cell lysates. Actin density blots were used as internal control. The representative immunoblot is from one of 5 independent experiments (B) Quantitative results expression is the mean ± S.E.M. of 5 independent experiments. (DOCX) [file pone.0102622.s003.docx]

**Figure S4**

**
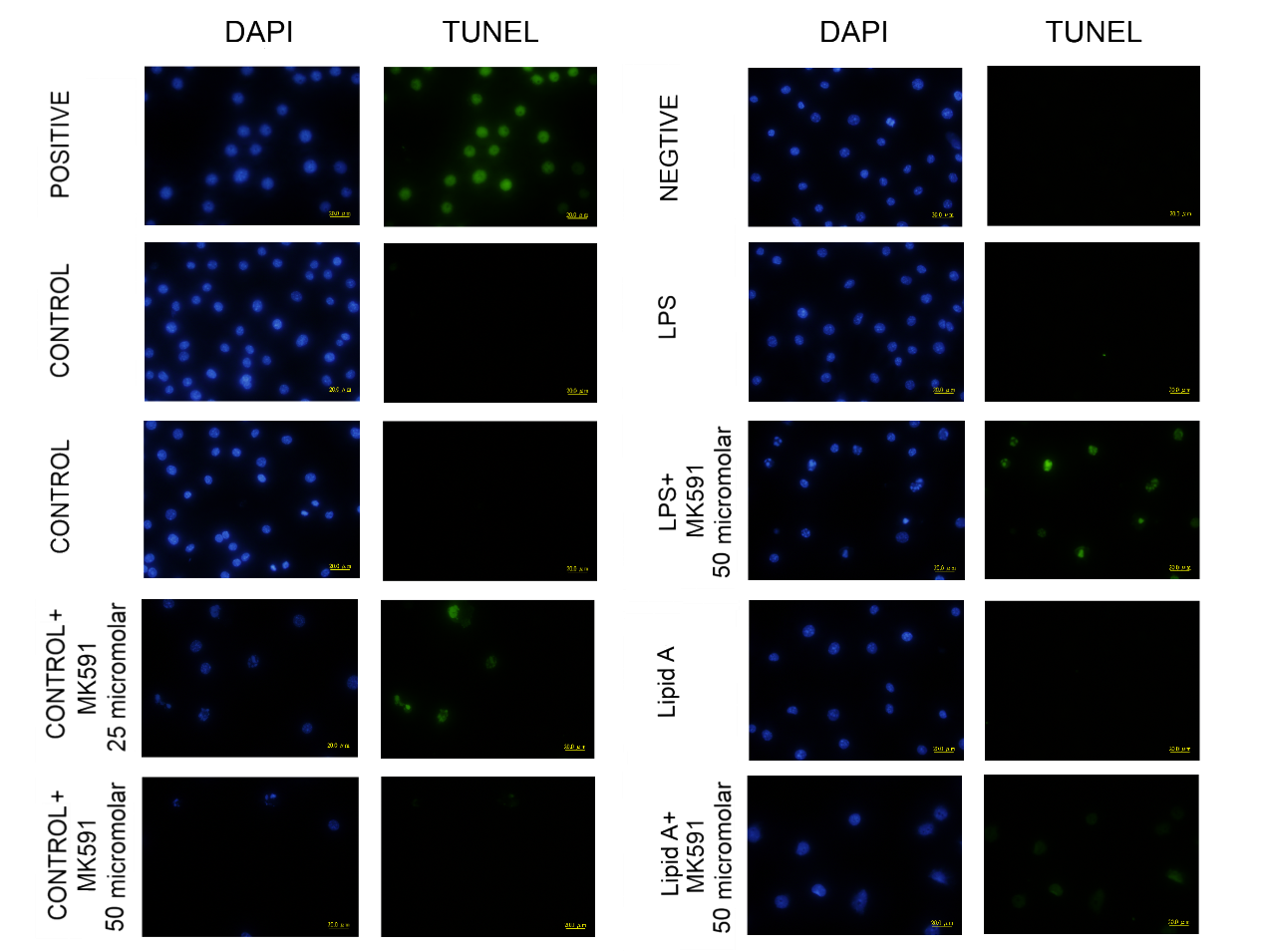
**

Supplement: Figure S4 — To further determine the effect of MK591 on murine macrophage proliferation and determine if cell death is an important contributor, the presence of apoptosis was measured by TUNEL assay. MK591 induced murine macrophages apoptosis with or without exposure to Lipid A or LPS. (DOCX) [file pone.0102622.s004.docx]

**Figure S5A**


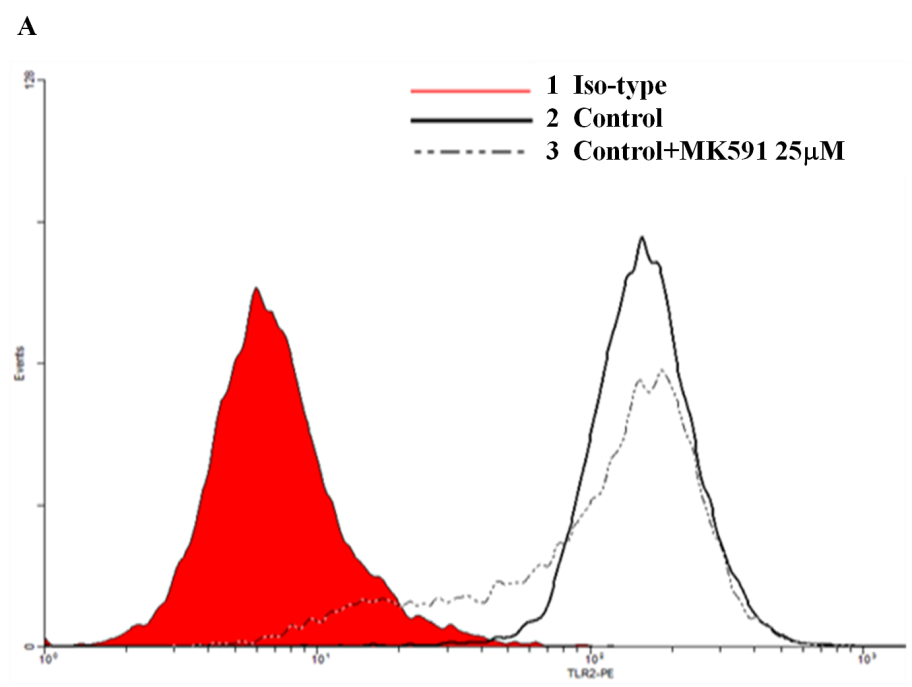


**Figure S5B**


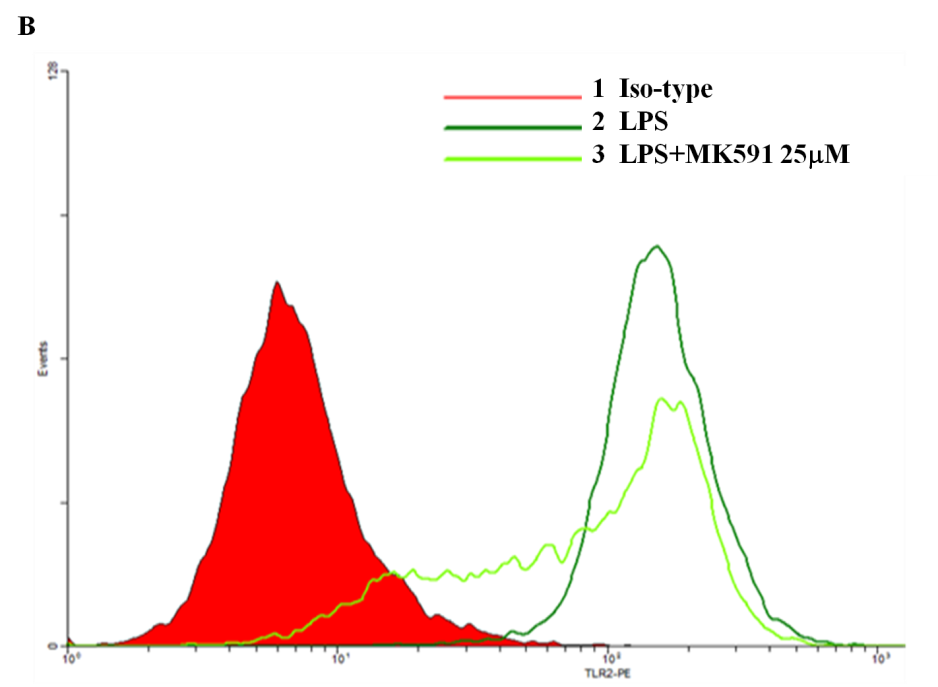


**Figure S5C**


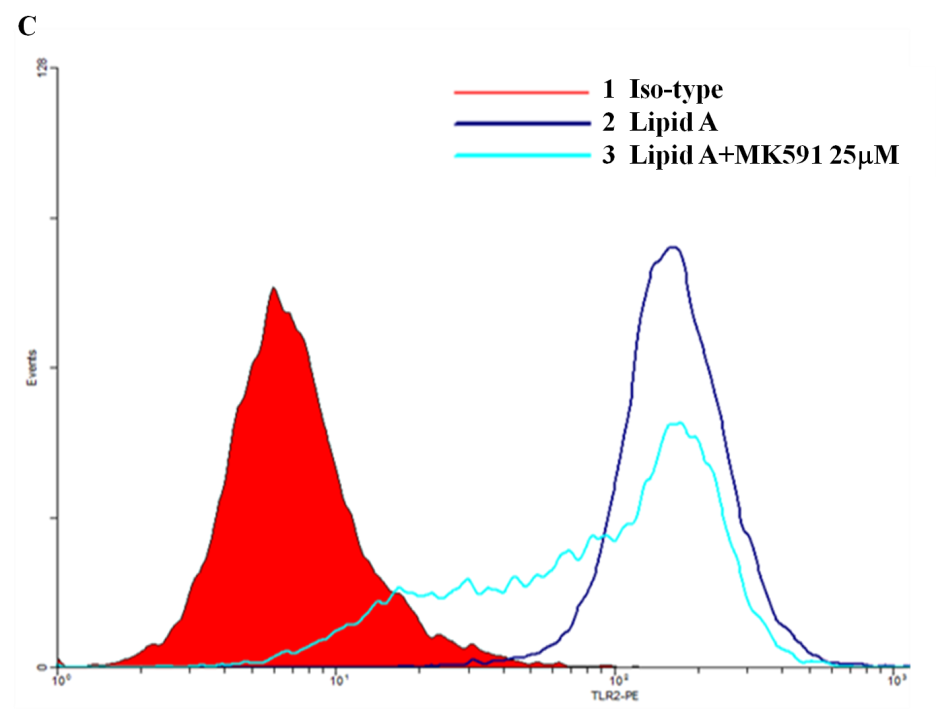


**Figure S5D**


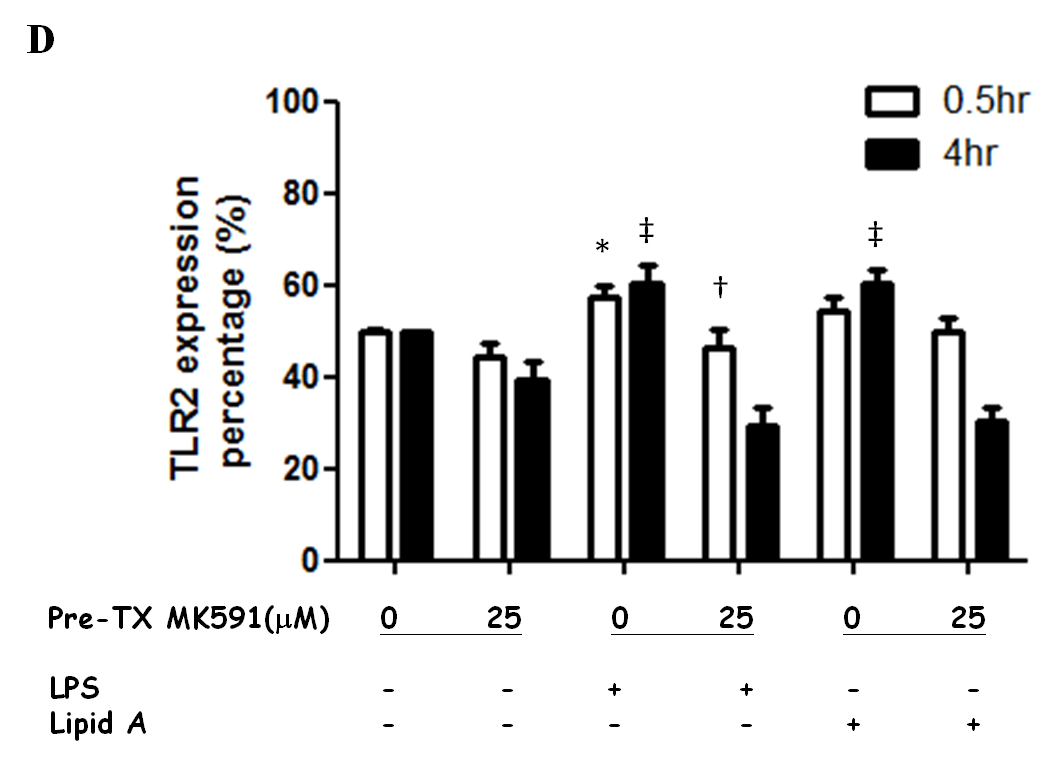


**Figure S5E**


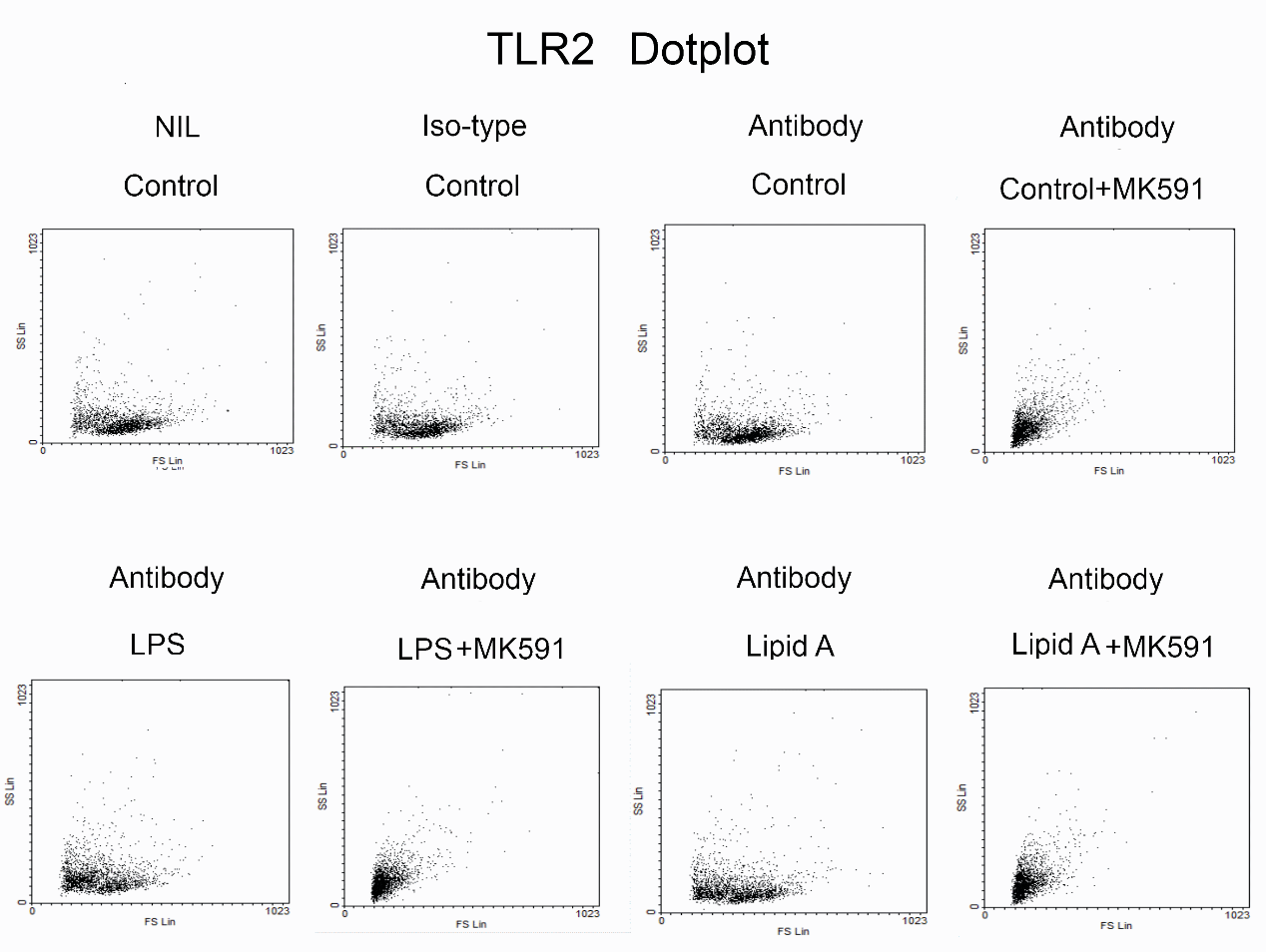

Supplement: Figure S5 — MK591 pre-treatment inhibits LPS or Lipid A-induced TLR2 expression on RAW264.7 cell membranes. (A) (B) (C) Flow cytometry histograms demonstrate reduced TLR2 expression after MK591 pre-treatment and LPS or Lipid A stimulation for 4 hours; expression is gated by the peak of control group. Shaded histogram represents isotype-matched negative control Ab fluorescence; open histogram represents specific Ab staining. (D) LPS and Lipid A treatment induces surface TLR2 expression, but this was inhibited by MK591 pretreatment. TLR2 expression quantitatively represented as mean ± S.E.M. of 6–7 independent experiments. At 0.5 hr, *p<0.05 compared to non-treated control cells and †p<0.05 compared to LPS-treated cells; as at 4 hr, ‡p<0.05 compared to non-treated control cells, §p<0.001 compared to LPS-treated cells and ¶p<0.001 compared to Lipid A-treated cells. (E) Representative flow cytometry dotplot demonstrate RAW264.7 cells after MK591 pre-treatment with LPS or Lipid A stimulation for 4 hours. (DOCX) [file pone.0102622.s005.docx]

**Figure S6A**


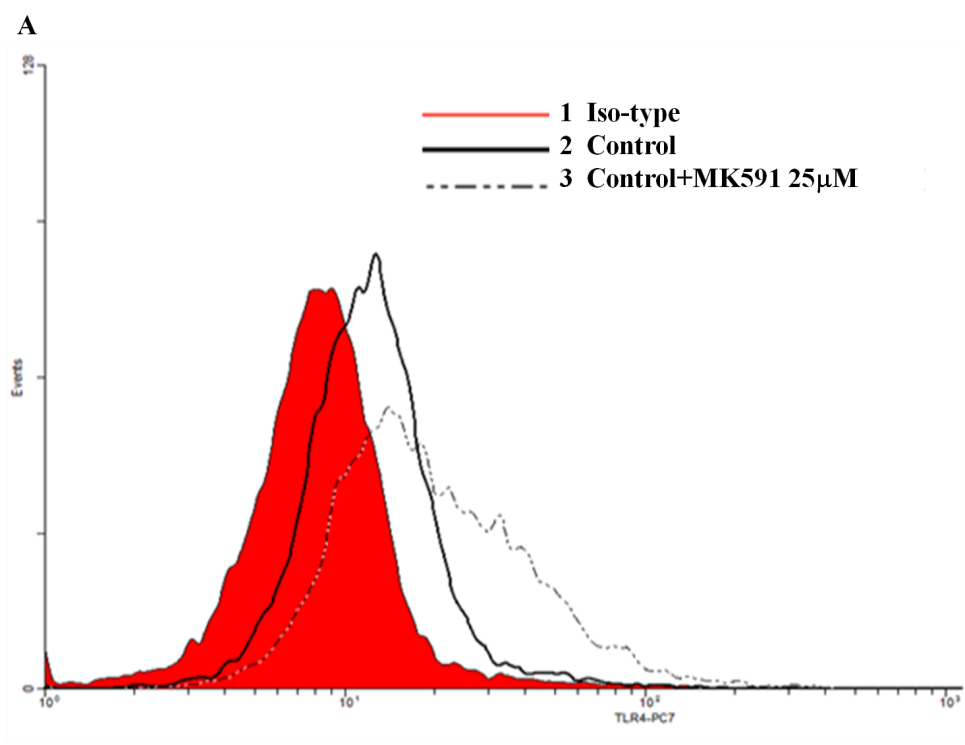


**Figure S6B**


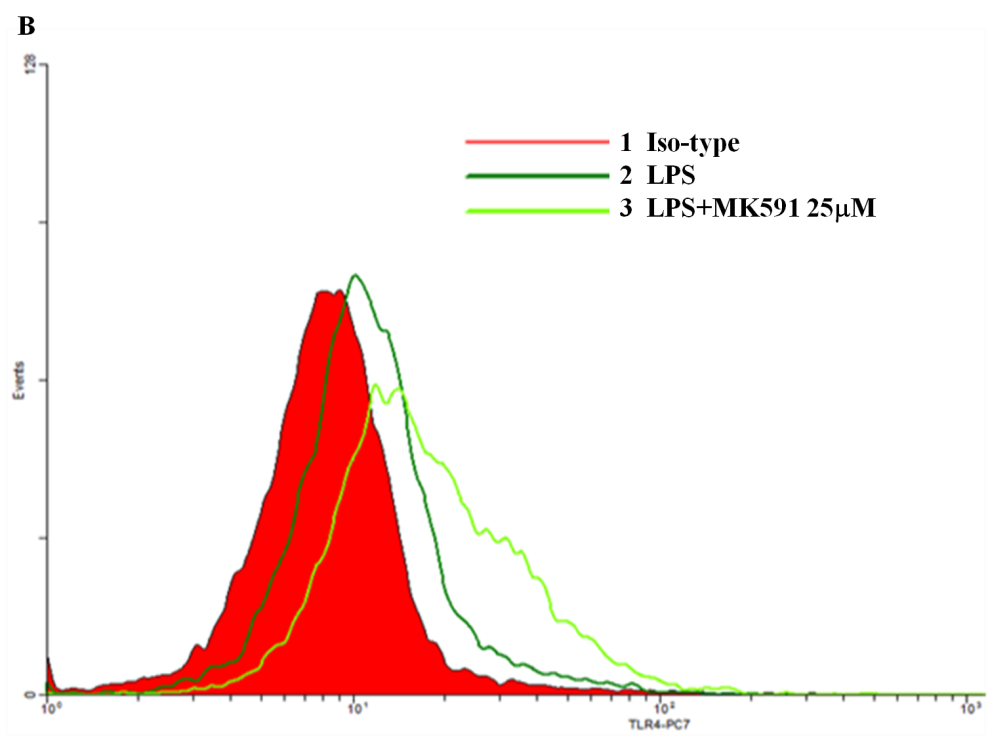


**Figure S6C**


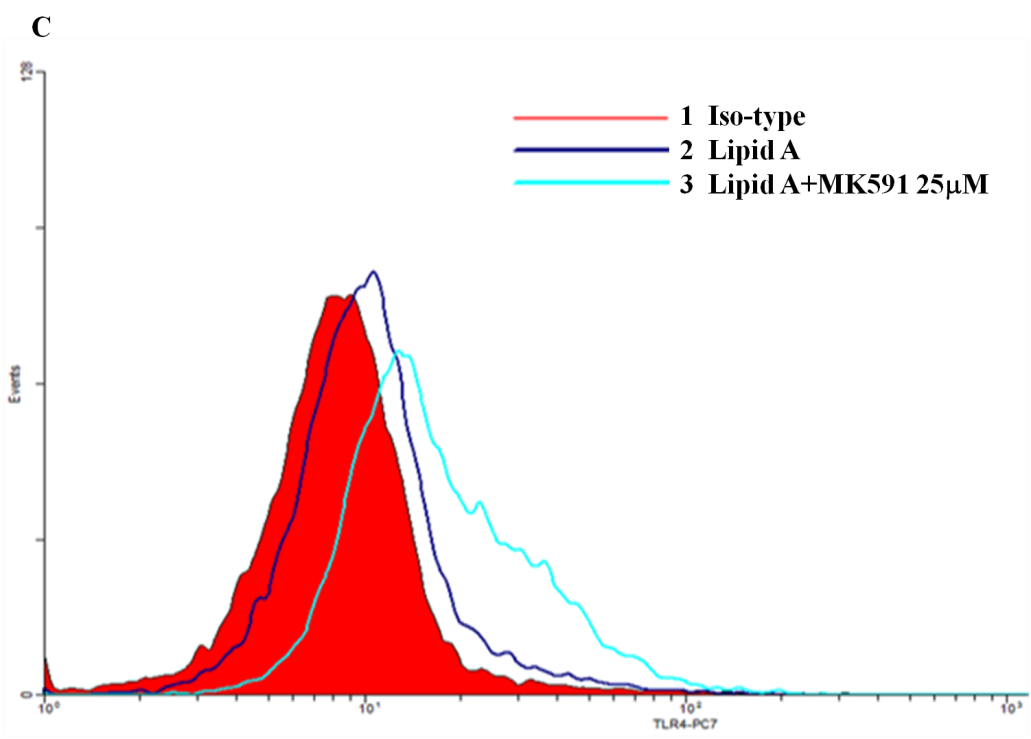


**Figure S6D**


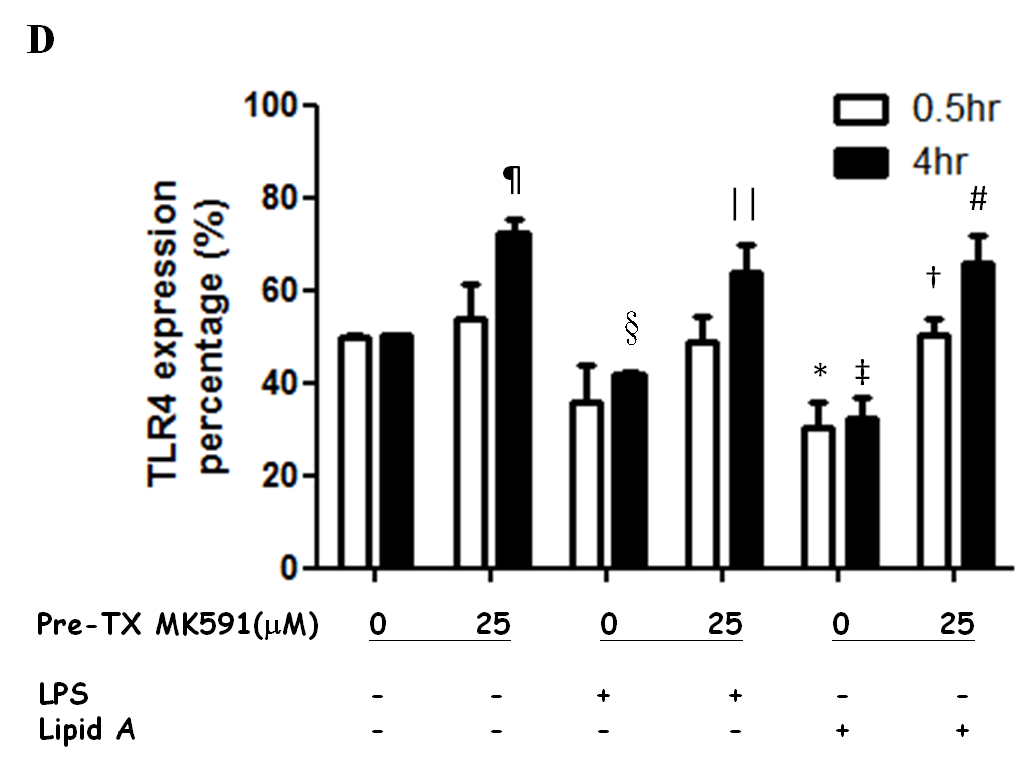


**Figure S6E**


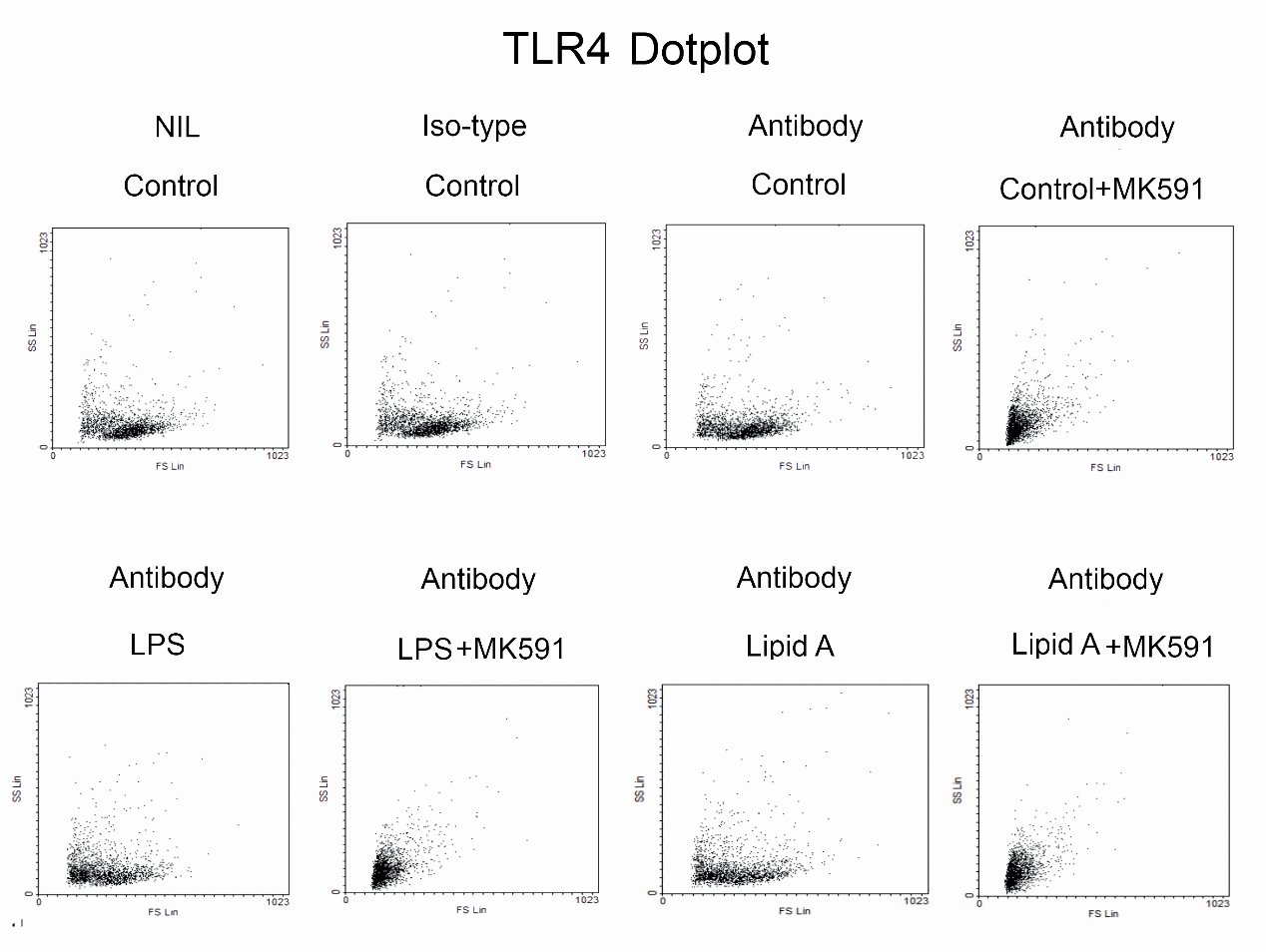

Supplement: Figure S6 — MK591 pre-treatment inhibits LPS or Lipid A-induced TLR4 expression on RAW264.7 cell membranes. (A) (B) (C) Flow cytometry histograms demonstrate increased TLR4 expression after MK591 pre-treatment and LPS or Lipid A stimulation for 4 hours; expression is gated by the peak of control group. Shaded histogram represents isotype-matched negative control Ab fluorescence; open histogram represents specific Ab staining. (D) LPS and Lipid A treatment reduced surface TLR4 expression, but this was reversed by MK591 pretreatment. Expression is quantitatively represented as the mean ± S.E.M. of 4 independent experiments. At 0.5 hr, *p<0.05 compared to non-treated control cells and †p<0.05 compared to Lipid A-treated cells; as at 4 hr, ‡p<0.05 and §p<0.001 compared to non-treated control cells, ¶p<0.01 compared to non-treated control cells, ||p<0.05 compared to LPS-treated cells and #p<0.01 compared to Lipid A-treated cells. (E) Representative flow cytometry dotplot demonstrate RAW264.7 cells after MK591 pre-treatment with LPS or Lipid A stimulation for 4 hours. (DOCX) [file pone.0102622.s006.docx]

**Figure S7.**


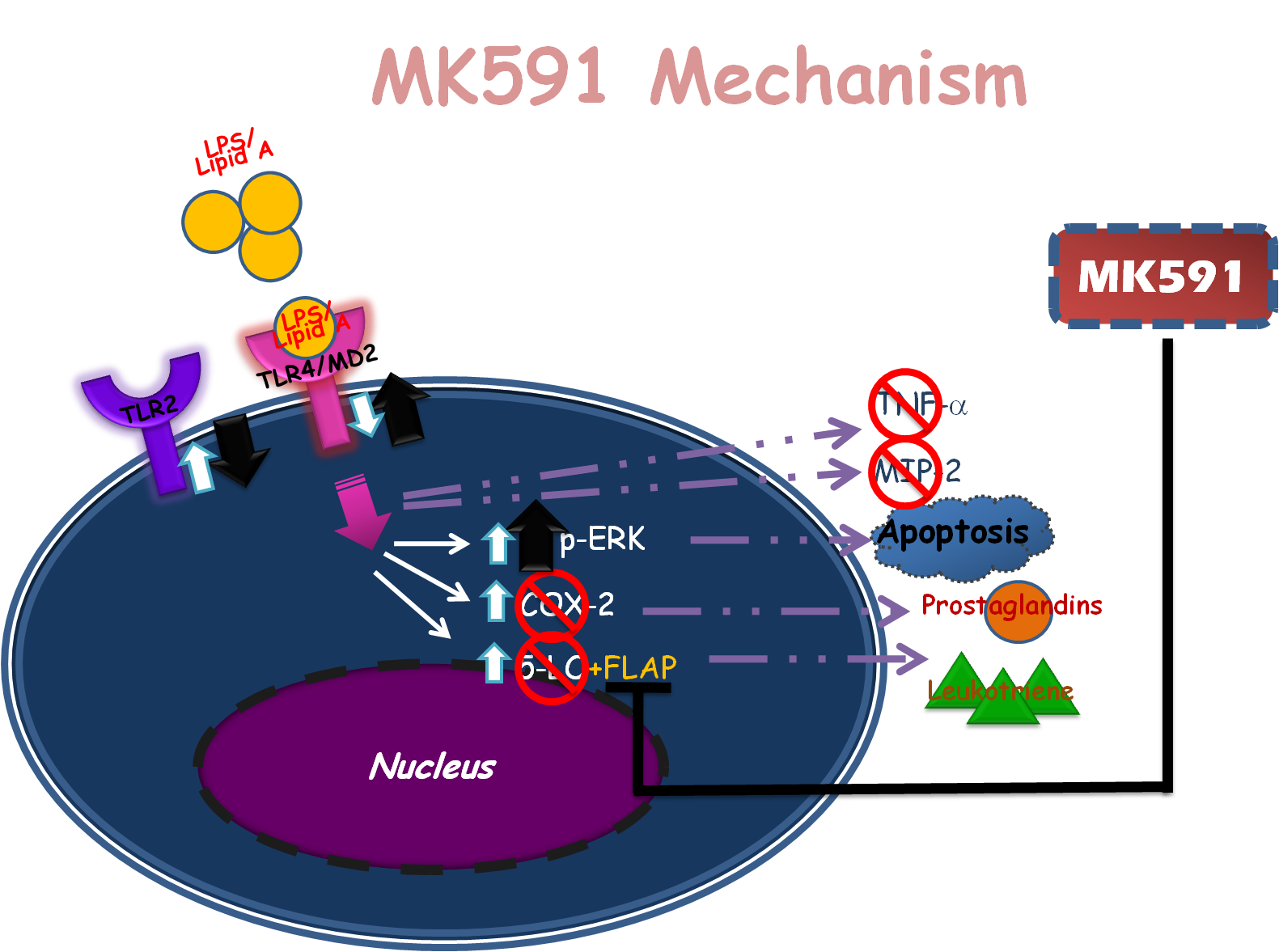

Supplement: Figure S7 — Model of LPS and Lipid A fraction regulation of inflammation by leukotriene biosynthesis inhibition. TLR4/MD2 engagement by LPS or the Lipid A fraction of endotoxin initiates macrophage-mediated inflammation (white arrows) with secretion of pro-inflammatory cytokines TNF-α and MIP 2, secretion of eicosanoid mediators and downregulation of cell-surface TLR4 expression. Leukotriene biosynthesis inhibition with MK591 (black arrows) block FLAP dependent 5-LO leukotriene biosynthesis, attenuates inflammation induced by either whole LPS or the Lipid A fraction of LPS in association with enhanced ERK activation and inhibition of cell proliferation. The likely mechanisms regulating this effect of leukotriene biosynthesis inhibition are increased apoptosis and modulation of cell surface receptor TLR expression. (DOCX) [file pone.0102622.s007.docx]
