# Supplementary material for: 5-Lipoxygenase Activating Protein (FLAP) Dependent Leukotriene Biosynthesis Inhibition (MK591) Attenuates Lipid A Endotoxin-Induced Inflammation
Source: PLoS One. 2014 Jul 15;9(7):e102622. doi: 10.1371/journal.pone.0102622 (PMC4099325; doi:10.1371/journal.pone.0102622)
Supplement: Methods S1 — Extended Materials and Methods. (DOCX) [file pone.0102622.s008.docx]

**Extended Materials and Methods**

**Method**

**Western blot analysis**

Western blots to detect levels of phosphorylated and total ERK, 5-lipoxygenase (5-LO), Cyclooxygenase-2 (COX-2) were performed. RAW264.7 cell after specific treatment were lysed in ice-cold lysis buffer (50mM HEPES, 50mM DTT, 1% Triton X-100, 10% Glycerol,pH 7.4) containing complete protease inhibitor cocktail (Roche Applied Science), then resuspended and sonicated for 30 seconds. Debris from the lysed cells was pelleted by centrifugation at 14,000rpm for 20 min. The supernatant was collected and stored at -86°C. The protein concentration of each sample was assayed using the Bio-Rad protein assay Dye (Bio-Rad) standardized to BSA, according to manufacturer’s protocol. 10 µg of protein was electrophered through a NuPAGE 4–12% Bis-Tris gel (Invitrogen) with a molecular weight marker. Proteins were electrotransferred to a polyvinylidene fluoride (PVDF, Immobilon-P; Millipore, Bedford, MA) membrane and then blocked with 5% nonfat dry milk, 20 mM TBS, with 0.1% Tween 20. After blocking, the membrane was incubated overnight at 4°C with a rabbit polyclonal specific primary antibody to phosphorylated ERK, 5-LO using a dilution of 1/1000 in 5% BSA, followed by anti-rabbit or anti-rat immunoglobulin HRP-coupled secondary antibody at a dilution of 1/3000 in 5% nonfat dry milk. After washing five times, bands were detected using ECL Western blotting detection reagents (Amersham Pharmacia Biotech, Piscataway, NJ). The membranes were stripped using stripping buffer (63 mM Tris-HCl, pH 6.8, 2% SDS, 100 mM 2-ME from Bio-Rad, Hercules, CA), and reprobed with antibodies specific for total ERK, COX-2 and β-Actin. Band density was measured using a chemiluminescence system running Quantity One software (Bio-Rad).

**In vitro apoptosis analysis**

The apoptotic effect of MK591 on murine macrophages was analyzed by using in situ terminal deoxynucleotidyl transferase dUTP nick end labeling (TUNEL) staining. 1×10^5^ cells were seeded in 12mm slides in 24-well plates in 1% FBS DMEM and incubated in 37°C overnight. The intervention groups were pre-treated with leukotriene biosynthesis inhibitor, 5-LO activated protein inhibitor (MK591), at a final concentration of 0, 10, 25, 50 and 100µM, respectively 30 minutes prior to stimulation. LPS and Lipid A will be added to induce inflammation at a final concentration of 100ng/ml and incubation for1 hr. The presence of apoptosis was assayed with the In situ Cell Death Detection, Fluorescein kit (Roche Diagnostics, Mannheim, Germany), following the manufacturer’s instructions. Additionally, the presence of cell nucleus was analyzed using 4,6-diamidino-2-phenylindole (DAPI) staining. The cells after *in situ* TUNEL staining were washed with PBS, and DAPI (Sigma Chemicals) was added (final concentration 0.2μg/ml) for 10 min at room temperature. The cells were washed two more times with PBS and were analyzed via fluorescence microscopy. Two staining control were prepared. Positive control were treated with 400U DNase Ⅰ/ml for 15 min at 37℃ before TUNEL staining and negative control TUNEL staining with only Label solution (without terminal transferase).
